# *Clostridium difficile* carriage in hospitalized cancer patients: a prospective investigation in eastern China

**DOI:** 10.1186/1471-2334-14-523

**Published:** 2014-09-29

**Authors:** Wei-Jia Fang, Da-Zhi Jing, Yun Luo, Cai-Yun Fu, Peng Zhao, Jiong Qian, Bing-Ru Tian, Xiao-Gang Chen, Yu-Long Zheng, Yi Zheng, Jing Deng, Wei-Hua Zou, Xue-Ren Feng, Fan-Long Liu, Xiao-Zhou Mou, Shu-Sen Zheng

**Affiliations:** First Affiliated Hospital, School of Medicine, ZheJiang University, 79 Qinchun Road, Hangzhou, 310006 China; Department of Microbiology, Zhejiang Provincial Center for Disease Control and Prevention, Hangzhou, China; Lab of Proteomics & Molecular Enzymology, School of Life Sciences, Zhejiang Sci-Tech University, Hangzhou, 310018 China; Department of Oncology, Yuyao Hospital, Yuyao, China; Huzhou Central Hospital, Huzhou, China; Zhejiang Provincial People’s Hospital, Hangzhou, China

**Keywords:** *Clostridium difficile*, Carriage, Age, Hospitalization days

## Abstract

**Background:**

*Clostridium difficile* carriage has been considered as a potential source for the deadly infection, but its role in cancer patients is still unclear. We aimed to identify the clinical and immunological factors that are related to *C. difficile* carriage in Chinese cancer patients.

**Methods:**

A total of 400 stool samples were collected from cancer patients who received chemotherapy in three hospitals of eastern China. Bacterial genomic DNA was extracted and two toxin genes (*tcdA and tcdB*) were detected. PCR ribotyping was performed using capillary gel electrophoresis. Concentrations of prostaglandin E2 (PGE2), transforming growth factor beta (TGF-β) and interleukin-10 (IL-10) were measured using enzyme-linked immunosorbent assay (ELISA) kits.

**Results:**

Eighty-two (20.5%) samples were confirmed to be *C. difficile*-positive and positive for tpi, *tcdA*, and *tcdB* genes. The *C. difficile*-positive rates in patients with diarrhea and no diarrhea were 35% and 19.7%, respectively (p = 0.09). Patients who were younger than 50 years old and were hospitalized for at least 10 days had a *C. difficile*-positive rate as high as 35%. In contrast, patients who were older than 50 years old and were hospitalized for less than 10 days had a *C. difficile*-positive rate of only 12.7% (p = 0.0009). No association was found between *C. difficile* carriage and chemotherapy regimen, antibiotic drug use, or immunosuppressive mediators, such as prostaglandin E2 (PGE2), transforming growth factor beta (TGF-β), or interleukin-10 (IL-10). Twelve ribotypes of *C. difficile* were identified, but none of them belonged to ribotype 027.

**Conclusions:**

We conclude that younger patients and those with longer hospitalization stays may be more prone to *C. difficile* carriage. Studies of larger populations are warranted to clarify the exact role of *C. difficile* carriage in hospitalized cancer patients in China.

**Electronic supplementary material:**

The online version of this article (doi:10.1186/1471-2334-14-523) contains supplementary material, which is available to authorized users.

## Background

*Clostridium difficile* infection is a hospital-acquired infection, and its prevalence has increased [1]. According to a report by the U.S. Centers for Disease Control and Prevention in 2013, *C. difficile* infection has been considered as an urgent threat and requires immediate attention. The major risk factors for *C. difficile* infection include the use of antibiotics, the use of proton pump inhibitors (PPIs), hospitalization, aging, and conditions that may affect the colonic flora [2–5].

It has been reported that cancer patients have a higher risk for *C. difficile* infection as compared to noncancer patients [6]. However, most information currently available on *C. difficile* infection is from the non-oncologic population. Until now, there is still a lack of comprehensive epidemiological studies of *C. difficile* infection in China, let alone specific investigations on cancer populations.

Herein, we conducted a comprehensive investigation on *C. difficile* carriage in cancer patients from three hospitals in eastern China. We hoped to identify potential clinical or laboratory factors that are related to *C. difficile* carriage in a variety of cancer patients.

## Methods

### Patients

Samples were collected from cancer centers in three hospitals (The First Affiliated Hospital of Zhejiang University, Yu-Yao Hospital, and Hu-Zhou Central Hospital). Each center has about sixty beds and they were expected to admit more than 1,000 cancer patients in total per year. All patients provided written consent, and the study protocol was approved by the Institutional Ethics Committees of all three hospitals, where samples were collected. The first stool sample after admission was collected from each cancer patient. Other information collected from the patients included the following: time of sample collection, age, gender, diagnosis, gastroenterological surgery within the last 3 months, cumulative days of hospitalization within the last 3 months, chemotherapy regimen and antibiotic drug use within the last 3 months, PPI use, diarrhea within the last 3 months, as well as counts of white blood cells and blood albumin. Medical records were reviewed and patient interviews were conducted to assess diarrhea symptoms.

Diarrhea was defined as having three or more loose, watery stool passages during a 24-h period [7, 8]. Diarrhea was further classified as follows: mild diarrhea, characterized by the absence of signs and symptoms of colitis; moderate diarrhea, characterized by the presence of colitis, fever, and abdominal cramps, usually in the lower quadrants; and severe diarrhea, defined as colitis associated with a leukocyte count greater than or equal to 15,000 cells/l and a serum creatinine level greater than or equal to 1.5 times the premorbid level [7, 8].

### Isolates from stool specimens and detection of toxin genes

All stool specimens were treated with alcohol, and the mixture was inoculated into cefoxitin-cycloserine fructose agar (CCFA) plates (Oxoid, Basingstoke) [9]. After incubation for 48 h at 37°C in a GENbag anaerobic chamber (BioMérieux, Marcy l’Etoile, France), the isolates were confirmed to be *C. difficile* based on assays as previously described [10]. Bacterial genomic DNA was extracted using a DNA extraction kit (Qiagen, Inc., Valencia, CA), according to the manufacturer’s instructions. The housekeeping gene tpi and the two toxin genes (*tcdA and B*) were detected using assays as previously described [9, 11]*.* Amplified products were analyzed by electrophoresis with ethidium bromide. DNA sequencing of all polymerase chain reaction (PCR) products was performed according to standard protocols.

### PCR ribotyping

PCR ribotyping was performed using capillary gel electrophoresis with primer pairs as described previously [12]. The size of each peak was determined using Genemapper ID-X software 1.3 (Applied Biosystems). The capillary sequencer-based PCR-ribotyping data were analyzed using the WEBRIBO website (https://webribo.ages.at/) [13].

### Detection of immunosuppressive mediators

Concentrations of prostaglandin E2 (PGE2; MaiBio, Shanghai, China), transforming growth factor beta (TGF-β; Ebioscience, San Diego, CA, USA), and interleukin-10 (IL-10; Ebioscience, San Diego, CA, USA) were measured using enzyme-linked immunosorbent assay (ELISA) kits. The ELISA kits were purchased from the above companies, respectively, and ELISA was carried out according to the manufactures’ instruction.

### Statistical analysis

**Figure 1:**
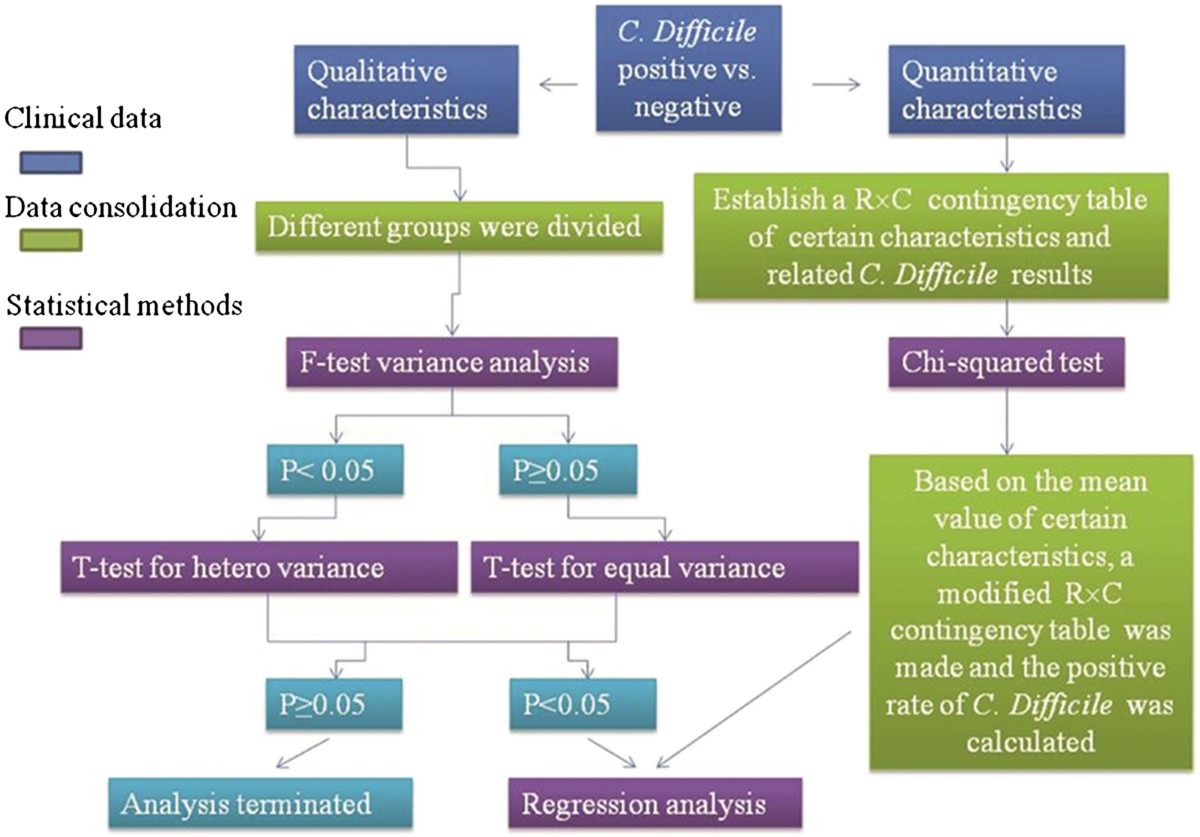
**Statistical analysis procedure.**

Statistical analysis was performed by SPSS 20.0 (Chicago, IL, USA). First, an R × C contingency table was established to consolidate the quantitative data. The data were then divided into different groups. The chi-squared test was used to analyze categorical data. The F-test and T-test were conducted for hetero or equal variance analysis. Significance of variables was determined by univariate Cox regression analysis (Figure 1). Statistical significance for all tests was defined as p < 0.05.

## Results

A total of 400 samples were collected from April to October, 2013. The numbers of samples obtained from Zhejiang University, Yu-Yao, and Hu-Zhou hospitals were 359, 26, and 15, respectively. There were 259 (65%) male and 141 (35%) female patients, and the median age was 57 years old. Further characteristics of the patients and *C. difficile* detection outcomes are listed in Table 1. Among the 82 (20.5%) *C. difficile*-positive patients, they were all *tcdA* and *tcdB* positive strains (*tcdA* + and *tcdB* +) and no toxin gene-deleted strains (*tcdA* + and *tcdB*-, *tcdA*- and *tcdB*+) were identified. All the 20 diarrheal patients were considered as mild diarrhea. No moderate or severe diarrhea was identified. The *C. difficile*-positive rates in the diarrhea and nondiarrhea patients were 35% and 19.7%, respectively (p = 0.09, as shown in Table 1). No association was found between the *C. difficile*-positive rate and clinical factors, including collection time, sex, diagnosis, PPI use, chemotherapy regimen and gastroenterological surgery within the last 3 months, and antibiotic drug use within the last 3 months.

**Table 1:** **Patient characteristics and** ***C. difficile*** **detection outcomes**

Category	Subdivision	Number (percentage, %)
Cancer type	Colorectal cancer	68 (17)
	Noncolorectal digestive tract cancer	128 (32)
	Nondigestive tract cancer	172 (43)
	Hematological malignancies	32 (8)
*C. difficile* Carriage	Positive	82 (20.5)
	Negative	318 (79.5)
Diarrhea	Yes	20 (5)
	*C. difficile-*positive	7 (35)^#^
	*C. difficile-*negative	13 (65)
	No	380 (95)
	*C. difficile-*positive	75 (19.7)^#^
	*C. difficile-*negative	305 (80.3)

^#^The *C. difficile*-positive rate was greater in diarrhea patients than in non-diarrhea patients; however, the difference was not statistically significant (p = 0.09).

### Age and *C. difficile*carriage

**Figure 2:**
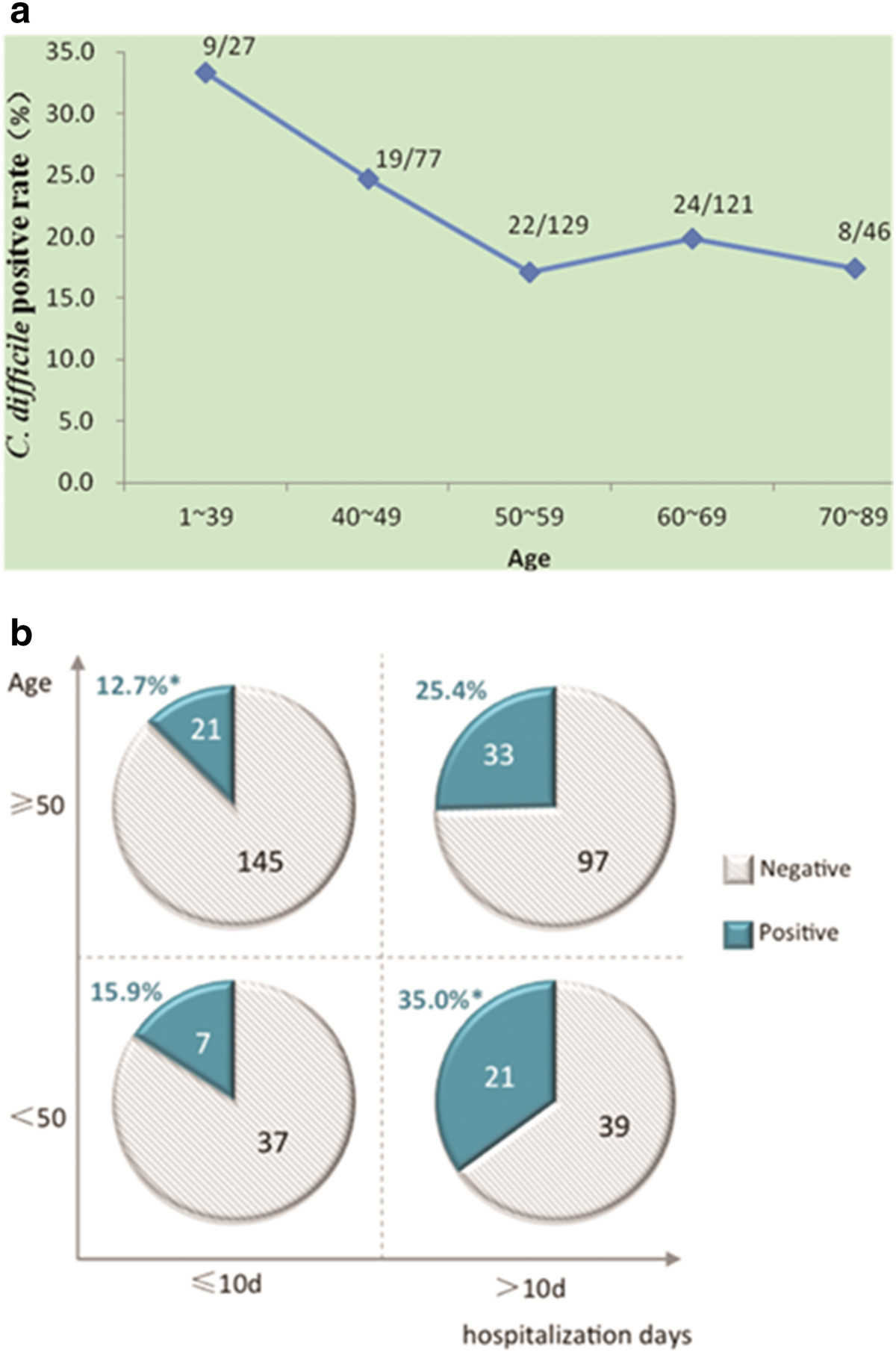
**Association of** ***C. difficile*** **positive rate with different age and hospitalization days. a**. Association between *C. difficile* positive rate and different age groups. Note: Patients were categorized into five groups of different ages, showing in the figure as positive number/total number of samples in each group. Patients younger than 50 years old had a significantly higher positive rate than the older groups (*p* = 0.04). **b**. Subdivision of *C. difficile* positive rate by age and days of hospitalization. Note: Patients were organized into two age groups by the cutoff value of 50 years old. Each age group was then subdivided into two groups based on the number of days in hospital (≤10 days or >10 days). *: The *C. difficile* positive rate was significantly different between the group that was <50 years old and hospitalized >10 days compared to the group that was ≥ 50 years old and hospitalized ≤10 days (35.0% vs. 12.7%, p = 0.0009).

The mean ages of the *C. difficile*-positive and *C. difficile*-negative patients were 54 and 56.7 years old, respectively. The T-test showed that the *C. difficile* infection rate between these two groups was not significantly different (p = 0.06). Next, the patients were divided into five consecutive age groups. As shown in Figure 2a, the cancer patients in the two age groups of patients who were younger than 50 years old had a higher infection rate than the other three groups of patients who were older than 50 years old. Therefore, 50 years old was set as the cut-off value and all of the patients were then grouped into two groups based on this age. The infection rate was 26.9% in the younger group and 18.2% in the older group. The difference was statistically significant (*x*^*2*^ = 4.17; p = 0.04).

### Cumulative days of hospitalization and *C. difficile*carriage rate

Next, it was determined if the cumulative number of days in hospital was related to the *C. difficile* positive rate. For the 318 *C. difficile*-negative patients, the cumulative number of days in hospital (within 3 months of sample collection) was 11.7 days. While for the *C. difficile*-positive patients, the cumulative number of days in hospital was 19.0 days, which was significantly longer (t = 3.5, p = 0.0005). Further analysis showed that the difference was more significant when all of the patients were divided into two groups: hospitalization for 10 days or longer and less than 10 days. The *C. difficile*-positive rates were 28.4% and 13.3%, respectively (*x*^*2*^ = 13.92; p = 0.0002). So, 10 days of hospitalization was set as the cut-off value for cancer patients.

### Combination of age, days of hospitalization, and *C. difficile*-positive rate

Using the two cut-off values of 50 years old and 10 days of hospitalization, the 400 cancer patients were divided into four groups and the positive rates of *C. difficile* in the various groups were analyzed. As shown in Figure 2b, the positive rate was as high as 35% in patients younger than 50 years old who were hospitalized for at least 10 days. In contrast, the positive rate was only 12.7% in patients older than 50 years old who were hospitalized for less than 10 days. The difference was highly significant (*x*^2^ = 16.5, p = 0.0009).

### PCR ribotyping of the positive strain

The ribotypes of the 82 *C. difficile* samples were determined. The results yielded 12 ribotype patterns, including ribotype 001 (n = 22), ribotype 017 (n = 15), ribotype 017/1 (n = 16), ribotype 014/0 (n = 12), ribotype 017/2 (n = 10), and one each of ribotypes 666, 650, 555, 445, 220, 037, and 087. None of the strains belonged to ribotype 027.

### Immunological indicators and *C. difficile*-positive rate

The albumin level, white blood cell count, and concentrations of PGE2, TGF-β, and IL-10 are important immunological indicators for cancer patients. These indicators were tested for their association with *C. difficile* carriage. All of the factors, including white blood cell count, albumin count, PGE2, TGF-β, and IL-10 concentrations, were not significantly different between the *C. difficile*-positive and negative patients.

## Discussion

In this study, we conducted a comprehensive investigation of *C. difficile* carriage among cancer patients from three hospitals in eastern China. The overall *C. difficile*-positive rate of the cancer patients enrolled in the 7-month period was 20.5%. This rate is much higher than that (12.6%) reported in non-cancer patients in a similar study in 2009 in eastern China [14]. The relationship between various clinical and immunological factors and *C. difficile* carriage was further analyzed. Two factors (age, days of hospitalization) were determined to be significantly associated with the rate of *C. difficile* carriage. First, we found that cancer patients with *C. difficile* carriage appeared to be younger than patients from the U.S. [15]. This difference could be caused by various factors, such as diet, geographical location, intake of food supplements and drugs, and other causes. Studies also have found that features of gut microbiomes are always unique to different locations and lifestyles [16]. The lifestyles of Chinese people, especially those of young Chinese, have changed dramatically over the last 20 years, partially because of the rapid growth of the Chinese economy. The changed lifestyle which was not so common in China in the past, might be a major contributor to the increasing emergence of *C. difficile* infection. Interestingly, the age predisposition was consistent with a previous study of cancer patients in Beijing, China [17]. Meanwhile, a large scale study in Japan also revealed that long hospital stay was associated with *C. difficile* infection [18]. However, these two studies did not provide a cutoff value for age and the hospitalization days. In our study, we found that the cancer patients who were younger than 50 years old and had stayed in hospital for more than 10 days were more prone to *C. difficile* carriage. It is easier to understand that longer stay in hospital exposes a higher risk of infection to patients; it is less clear to us why the younger patients in our study were more prone to *C. difficile* carriage. A similar result was reported recently in northern China [17].

Immune response is important for *C. difficile* carriage. However, among white blood cell count, albumin count, and levels of PGE2, TGF-β, and IL-10, which are thought to be inhibitors of immunological activity [19], we found no factor was related to the *C. difficile*-positive rate. And ribotyping of *C. difficile* showed that there was no 027 strain, which was the major cause of the deadly *C. difficile* infection emergence in Canada between 2002 and 2005 [20].

Strikingly, more than 90% of the *C. difficile*-positive cancer patients in our study had no symptoms of diarrhea. The reason might be complicated. A similar study also showed no outbreak of *C. difficile* infection despite *C. difficile* carriage [21]. Nevertheless, researchers have suggested that asymptomatic carriers can contribute to *C. difficile* transmission in hospitals [22]. As a result of the near-indefinite viability of the bacteria and the low infective dose, *C. difficile* could be widely transmitted within hospitals in the presence of the ever increasing asymptomatic carriers [23].

## Conclusions

In our current study, no cancer-specific factors were identified to be related to *C. difficile* carriage. However, a younger age and a longer hospitalization stay may represent the characteristics of more aggressive and immunosuppressive oncologic disease. Larger sample size and cancer-specific information mining research is needed in the future in order to clarify the exact role of *C. difficile* carriage in cancer patients.

## Authors’ original submitted files for images

Below are the links to the authors’ original submitted files for images. Authors’ original file for figure 1 Authors’ original file for figure 2 Authors’ original file for figure 3

## Abbreviations

*C. difficile*: *Clostridium difficile*
PGE2: Prostaglandin E2
IL-10: interleukin-10
TGF-β: Transforming growth factor beta
PPIs: Proton pump inhibitors
CCFA: Cefoxitin-cycloserine fructose agar
ELISA: Enzyme-linked immunosorbent assay.
