# Supplementary figures and images for: Clostridium difficile carriage in hospitalized cancer patients: a prospective investigation in eastern China
Source: BMC Infect Dis. 2014 Sep 29;14:523. doi: 10.1186/1471-2334-14-523 (PMC4261591; doi:10.1186/1471-2334-14-523)

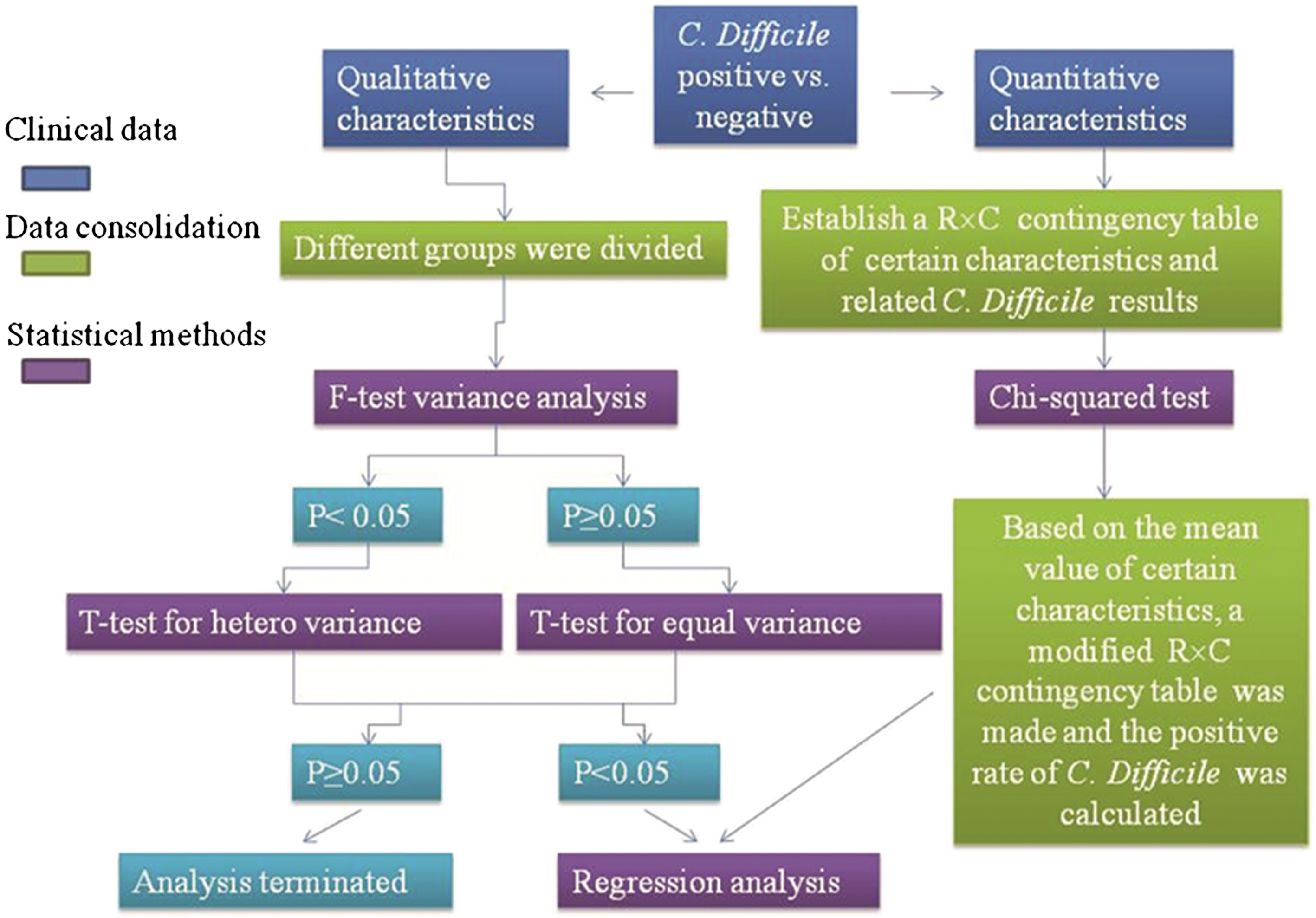

Supplement: Supplementary file 1 — Authors’ original file for figure 1 [file 12879_2014_3843_MOESM1_ESM.tif]

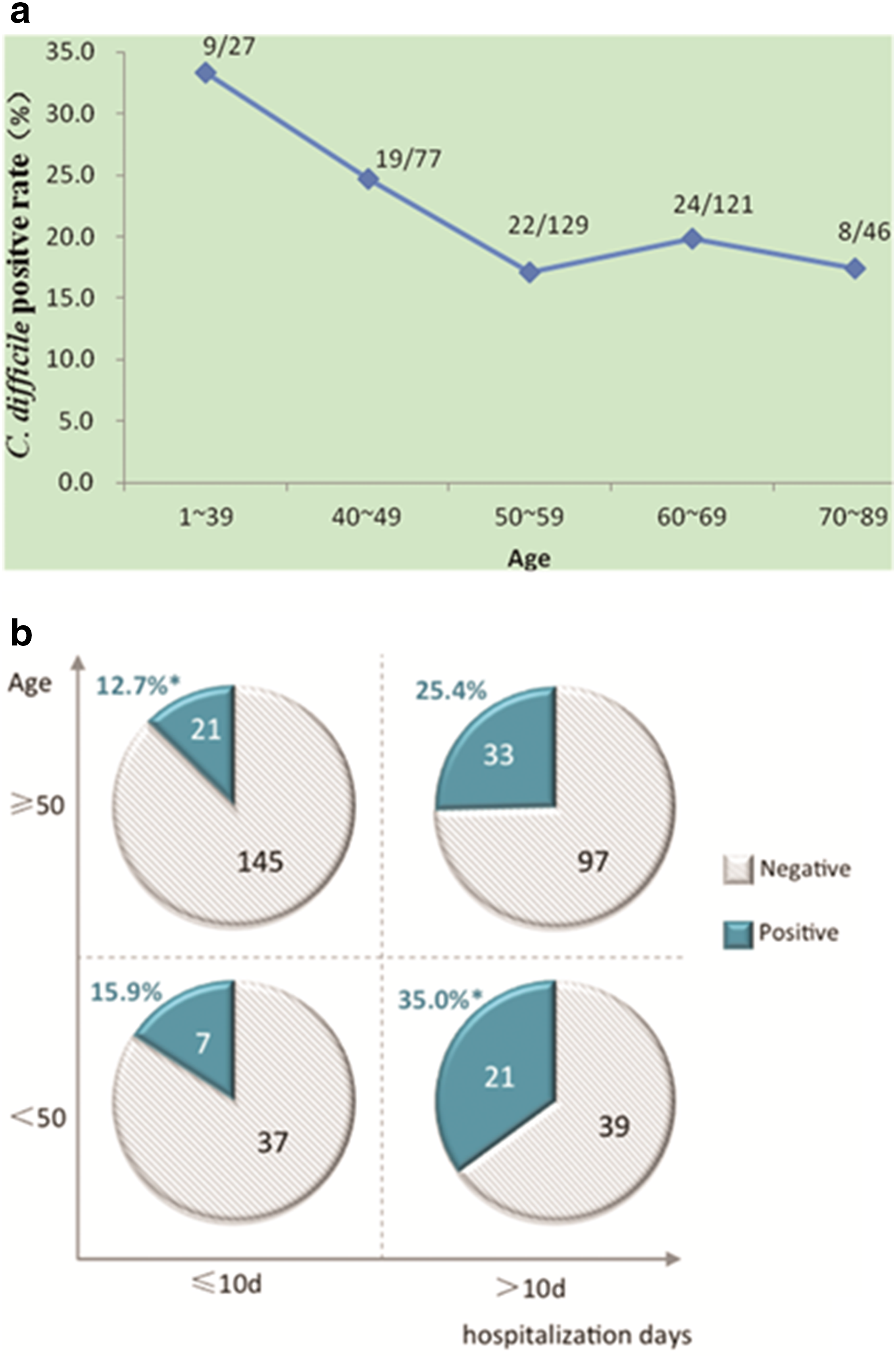

Supplement: Supplementary file 2 — Authors’ original file for figure 2 [file 12879_2014_3843_MOESM2_ESM.tif]

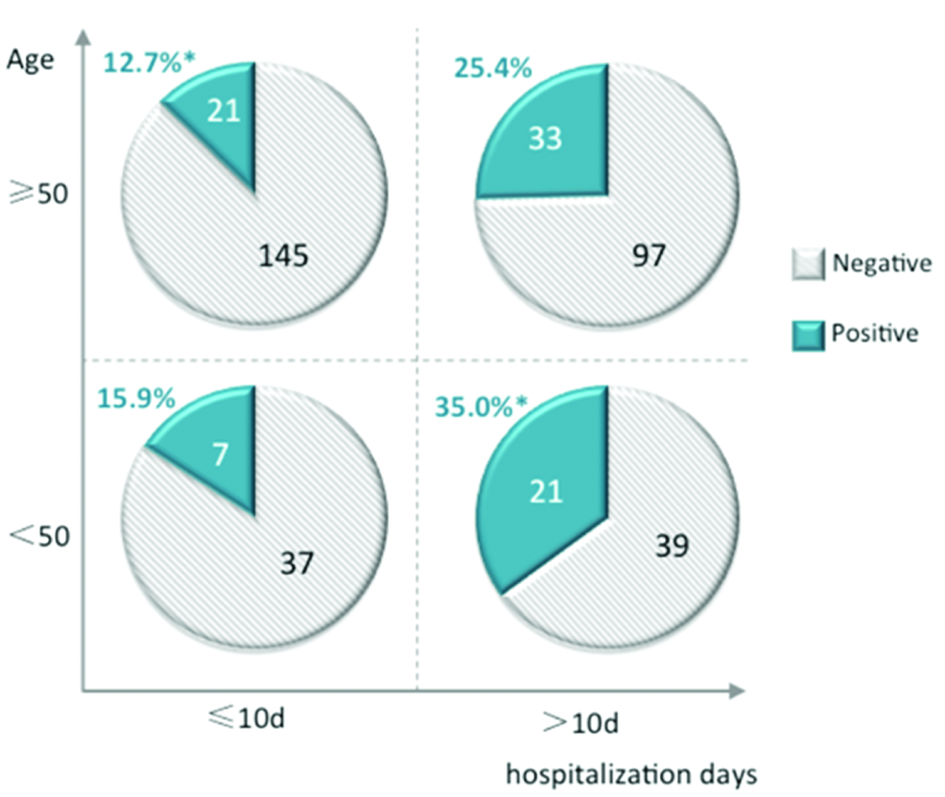

Supplement: Supplementary file 3 — Authors’ original file for figure 3 [file 12879_2014_3843_MOESM3_ESM.tiff]
